# Depression, Anxiety, and Other Mental Disorders in Patients With Cancer in Low- and Lower-Middle–Income Countries: A Systematic Review and Meta-Analysis

**DOI:** 10.1200/GO.21.00056

**Published:** 2021-08-03

**Authors:** Zoe J. Walker, Siqi Xue, Michael P. Jones, Arun V. Ravindran

**Affiliations:** ^1^ Department of Psychiatry, University of Toronto, Toronto, Ontario, Canada; ^2^ Centre for Addiction and Mental Health, Toronto, Ontario, Canada; ^3^ Royal Hobart Hospital, Hobart, Australia; ^4^ Department of Radiation Oncology, University of Toronto, Toronto, Ontario, Canada; ^5^ Princess Margaret Cancer Centre, Toronto, Toronto, Ontario, Canada

## Abstract

Cancer is a growing public health issue in low- and lower-middle–income countries (LLMICs), but the mental health consequences in this setting have not been well-characterized. We aimed to systematically evaluate the available literature on the prevalence, associates, and treatment of mental disorders in patients with cancer in LLMICs.

**METHODS:**

We systematically searched Medline, PsycINFO, EMBASE, and CINAHL. We performed a random effects meta-analysis to determine the pooled prevalence of major depression or anxiety disorders in this population, defined by Diagnostic and Statistical Manual of Mental Disorders or International Classification of Diseases criteria. We qualitatively reviewed studies that examined the prevalence of depressive or anxiety disorders defined by self-report tools, the prevalence of other mental disorders, associated factors of depressive and anxiety symptoms, and the treatment of mental disorders in this population.

**RESULTS:**

Forty studies spanning a 15-year period were included in the review. The pooled prevalence defined by Diagnostic and Statistical Manual of Mental Disorders or International Classification of Diseases criteria was 21% for major depression (95% CI, 15 to 28) and 18% for anxiety disorders (95% CI, 8 to 30). Depressive and anxiety symptoms were most frequently associated with advanced disease and low levels of education. Among the four studies evaluating treatment, three evaluated the effectiveness of psychotherapy and one evaluated a yoga program.

**CONCLUSION:**

The prevalence of depression and anxiety in patients with cancer generally appears higher in LLMICs than in upper-income countries. Our findings demonstrate the existence of a significant and underappreciated disease burden. We suggest that clinicians remain vigilant to psychiatric symptoms. Improved screening and treatment are likely to improve quality of life and reduce both morbidity and mortality.

## INTRODUCTION

Cancer is currently the second leading cause of death worldwide, and the global burden continues to grow.^1^ Between 2008 and 2030, the global incidence is expected to increase by more than 80%, with the greatest increases predicted to occur in less-developed countries.^2^ Literature from developed countries clearly confirms that patients with cancer have higher rates of depression and anxiety than the general population^3-5^ and that cancer comorbidity with depression results in greater morbidity and poorer cancer-related outcomes.^6,7^

**CONTEXT**

**Key Objective**
To describe the intersection between cancer and mental disorders in low- and lower-middle–income countries, which has not been systematically evaluated previously.
**Knowledge Generated**
The prevalence of interview-defined major depression was 21%, and the prevalence of interview-defined anxiety disorder was 18%. Depressive and anxiety symptoms were most frequently associated with advanced disease and low levels of education.
**Relevance**
This review establishes the existence of a significant disease burden. It highlights the importance of mental disorder symptom recognition and treatment. It lends further support to the integration of mental health services within cancer care centers in LLMICs.

Compared with upper-income countries, patients with cancer in low- and lower-middle–income countries (LLMICs) are generally diagnosed at a more advanced stage, have limited access to treatment, and face a poorer prognosis.^8,9^ Although almost half the world's population resides in LLMICs,^10^ the mental health burden among patients with cancer in LLMICs has not been systematically evaluated. Many LLMICs are in the process of developing comprehensive cancer care programs. The International Federation of Psycho-Oncology Societies advocates for the integration of psychosocial support into routine cancer care.^11^ Quantifying and characterizing the burden of mental disorders in this population may inform the development of cancer care services in LLMICs.

To our knowledge, no review has comprehensively examined the intersection between cancer and mental disorders in LLMICs. Thus, within this population, the aims were to

1. Systematically review the prevalence of depression, anxiety, and other mental disorders and perform a pooled prevalence meta-analysis where appropriate
2. Systematically review factors affecting mental disorder symptoms
3. Systematically review the effectiveness of treatment.

## METHODS

### Protocol and Registration

The meta-analysis followed Preferred Reporting Items for Systematic Reviews and Meta-Analyses (PRISMA) guidelines. It was registered with PROSPERO (CRD42017057103), and the protocol was published.^12^

### Search Strategy and Study Selection

The protocol contains a detailed description of the search strategy and the eligibility criteria for inclusion. In brief, the search used a comprehensive list of subject headings and keywords to link the broad concepts of (1) neoplasms, (2) LLMICs, and (3) mental disorders. The specific search strategies for MEDLINE, PsycInfo, EMBASE, and CINAHL are shown in the Data Supplement. MEDLINE, PsycInfo, EMBASE, and CINAHL were searched for all studies that met the inclusion criteria. The search was performed on March 31, 2017. Two additional searches were performed on July 16, 2017: one search to reflect the addition of three countries to the World Bank's 2018 fiscal year list of LLMICs, released on July 1, 2017, and another to capture eligible articles published since the initial search.

The target population was adults with cancer living in LLMICs, as defined by the World Bank's 2018 fiscal year list. Articles were included if they had been published in English after March 2002 and reported original peer-reviewed data on either the prevalence of mental disorders or the outcome of interventions addressing depression or anxiety in the target population. Validation studies and studies examining prevalence with fewer than 20 participants were excluded. Where necessary, study authors were contacted for data or clarification. Where there were multiple reports on the basis of the same study population, the results were taken from the study reporting the largest sample. The reference lists of included studies were also screened for any missed publications.

### Data Management and Study Selection

The literature search results were uploaded to Endnote X8. Z.J.W. and M.P.J. independently screened the titles and abstracts against the inclusion criteria and created a preliminary list of articles. The full text was retrieved, and Z.J.W. and M.P.J. jointly screened the full text of these articles. Disagreements were resolved through consensus. The reasons for exclusion were documented in the PRISMA flow diagram. Z.J.W. extracted the primary data from the included studies into both a Word document table and Excel spreadsheets. The data were cross-checked by S.X.

### Risk of Bias

Z.J.W. and S.X. independently rated the quality of individual studies using the National Institute of Health's Study Quality Assessment Tools.^13^ Each study was rated as being good, fair, or poor, which equated to being at a low, medium, or high risk of bias, respectively. Disagreements were resolved by consensus. The quality assessment charts are given in the Data Supplement. Risk of publication bias across all studies was assessed using funnel plots. To minimize classification bias, studies were separated into two categories: those that defined major depression or anxiety according to either Diagnostic and Statistical Manual of Mental Disorders, Fourth Edition (DSM-IV) or International Classification of Diseases 10th Revision (ICD-10) criteria using interview-based tools and those that defined depression or anxiety caseness using self-report tools. Both categories were analyzed and reviewed separately.

### Statistical Analysis

A pooled prevalence meta-analysis was performed for major depression defined by interview-based tools and for anxiety disorders defined by interview-based tools. Raw counts were used in the analysis. In rare cases, when only proportions were available, they were converted into a raw count. For longitudinal studies, baseline prevalence values were used in the analysis. There were insufficient numbers for valid subgroup analyses on the basis of outcome measurement instrument, World Bank region, or country. All statistical analyses were programmed using the metaprop command in Stata (version 14). Meta-analyses used random effects modeling and Freeman-Tukey Double arcsine transformation. Sensitivity analyses were conducted, excluding studies that were identified as being at a high risk of bias.

### Qualitative Review

A narrative synthesis was performed for the prevalence of depression and anxiety defined by self-report tools and the prevalence of other mental disorders. Statistically significant associated factors of depressive and anxiety symptoms identified by prevalence studies were also synthesized, as were those studies examining the effectiveness of interventions.

### Role of the Funding Source

The NSWIOP had no role in study design, data collection, data analysis, data interpretation, or writing of the report.

## RESULTS

**FIG 1:**
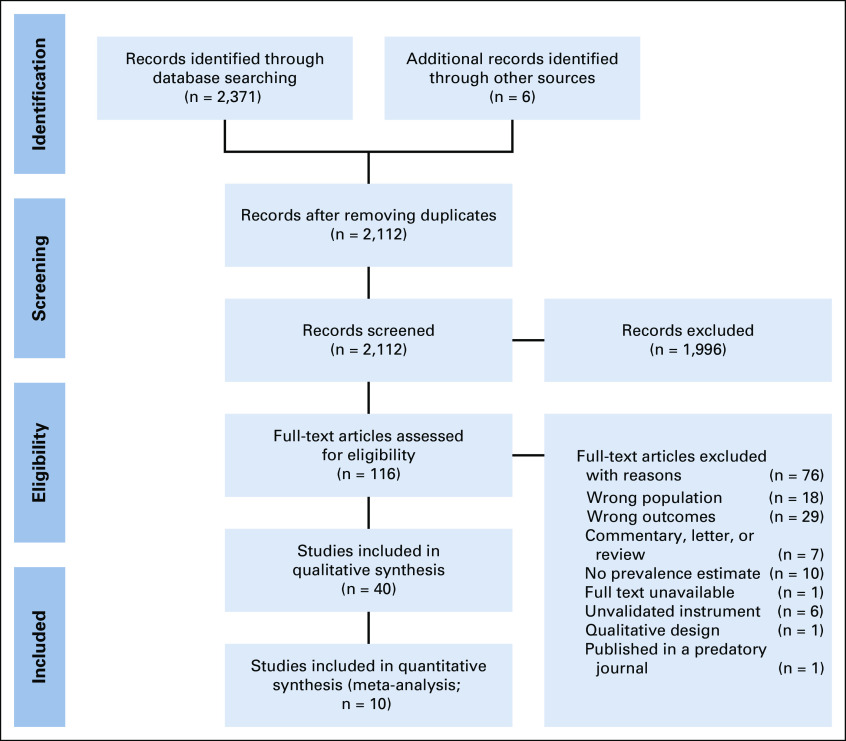
Preferred reporting items for systematic reviews and meta-analyses flowchart.

The literature search returned 2,371 records published in the English language between 2002 and 2017, with six additional reports found through other sources. Of the 116 full-text articles screened, 40 met the inclusion criteria. The PRISMA flowchart is shown in Figure 1. The majority (26 of 40) of studies were published after 2012. The articles contained data from 15 countries spanning four World Bank regions: East Asia and Pacific, South Asia, Middle East and North Africa, and Sub-Saharan Africa. Nigeria and India had the most frequent representation with 10 and eight articles from these countries, respectively. Cancer type was often reported as mixed; however, 12 studies limited their study populations to patients diagnosed with breast cancer.

There were 36 studies identified reporting prevalence data on 32 separate study populations. The extracted study characteristics, prevalence data, study quality, and significantly associated factors are given in Table 1. Of these 36 studies, 30 contained data on associated factors. Four interventional studies were identified. The prevalence studies contained data on 9,195 participants in total, with 5,637 participants in studies of associated factors. Most studies were cross-sectional and defined depressive or anxiety disorders using clinical interviews or self-report tools. Four interventional studies were identified, involving a total of 217 participants. Overall, 13 prevalence studies were classified as being at low risk of bias, 18 at medium risk of bias, and five at high risk of bias, whereas two of the interventional studies were classified as being at a medium risk of bias and two at a high risk of bias. The most common study weaknesses are related to poor sampling methodology or poor reporting.

**TABLE 1:**
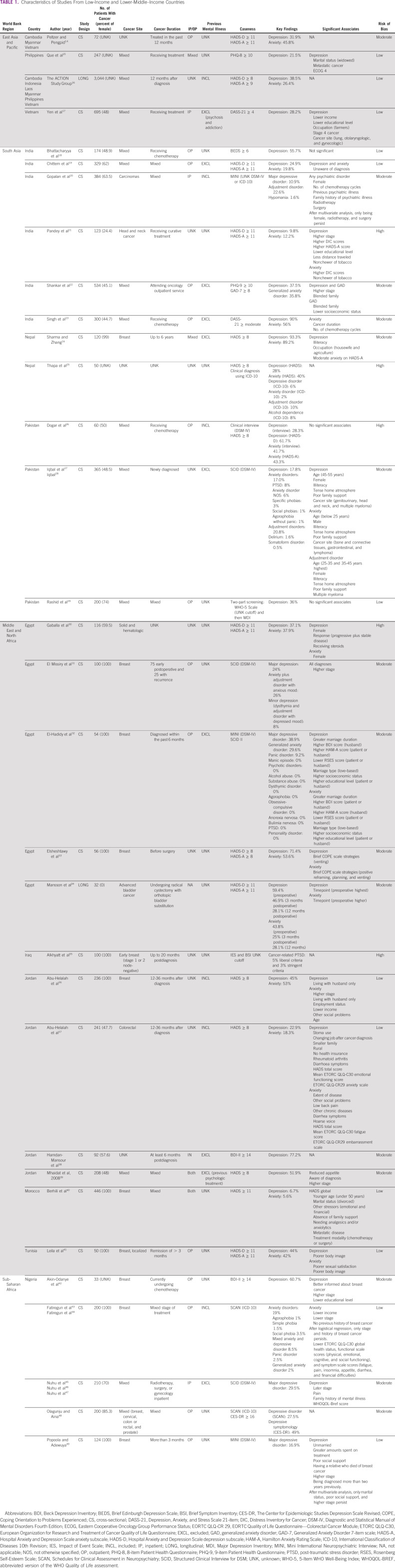
Characteristics of Studies From Low-Income and Lower-Middle–Income Countries

**FIG 2:**
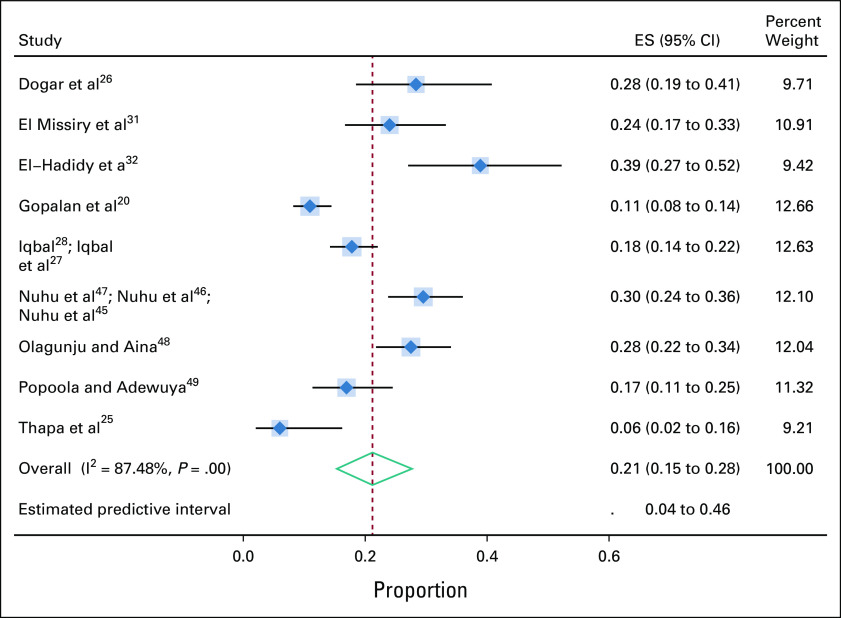
Prevalence of major depression (interview-based tools). ES, estimate.

For major depression defined by interview-based tools on the basis of either DSM-IV or ICD-10 criteria, 12 studies reported prevalence estimates involving 1,547 participants drawn from nine separate study populations. The diagnostic criteria for major depression as per DSM-IV and ICD-10 are included in the Data Supplement. The pooled prevalence of major depression as defined by interview-based tools was 21% (95% CI, 15 to 28). The I-squared value was 87.5%, indicating a high degree of study heterogeneity (Fig 2). Sensitivity analysis yielded no substantial change. There was no evidence of publication bias.

**FIG 3:**
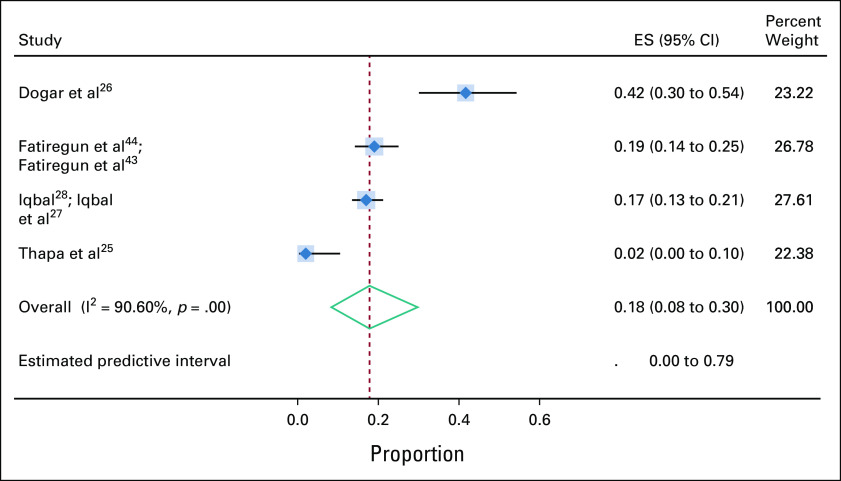
Prevalence of anxiety disorders (interview-based tools). ES, estimate.

Six studies reported prevalence estimates of DSM-IV–defined or ICD-10–defined anxiety disorders identified by interview-based tools. The six studies were drawn from four separate study populations involving 675 participants. The pooled prevalence of anxiety disorders defined by interview-based tools was 18% (95% CI, 8 to 30). The I-squared value was 90.6%, indicating high heterogeneity (Fig 3). Sensitivity analysis produced a marginal change. There was no evidence of publication bias.

Several studies defined depression and anxiety caseness using self-report tools. A range of self-report tools and cutoff scores were used, resulting in highly variable prevalence estimates. The Hospital Anxiety and Depression Scale (HADS) was the most frequently used instrument.

For depression defined by self-report tools, 24 studies reported prevalence estimates on 7,657 participants, with the range spanning 6.7%^40^-93.3%.^24^ Eight studies used a HADS subscale cutoff of ≥ 11, whereas seven used an HADS subscale cutoff of ≥ 8. Prevalence estimates of anxiety defined by self-report tools were reported by 15 studies involving 5,275 participants, with the range spanning 5.6%^40^-89.2%.^24^ Seven studies used an HADS subscale cutoff of ≥ 11, and six used an HADS subscale cutoff of ≥ 8, whereas one used an HADS subscale cutoff of ≥ 9. For both depression and anxiety, the lowest estimates were reported in a Moroccan sample of women with breast cancer using an HADS subscale cutoff of ≥ 11,^40^ whereas the highest estimates came from a Nepalese sample of women with breast cancer using an HADS subscale cutoff of ≥ 8.^24^

The prevalence of other mental disorders is summarized in Table 2. Generally, there were few reports, with Adjustment Disorder, Generalized Anxiety Disorder, Agoraphobia, and Post-Traumatic Stress Disorder (PTSD) each reported three times. A variety of interview-based tools and self-report tools were used.

**TABLE 2:**
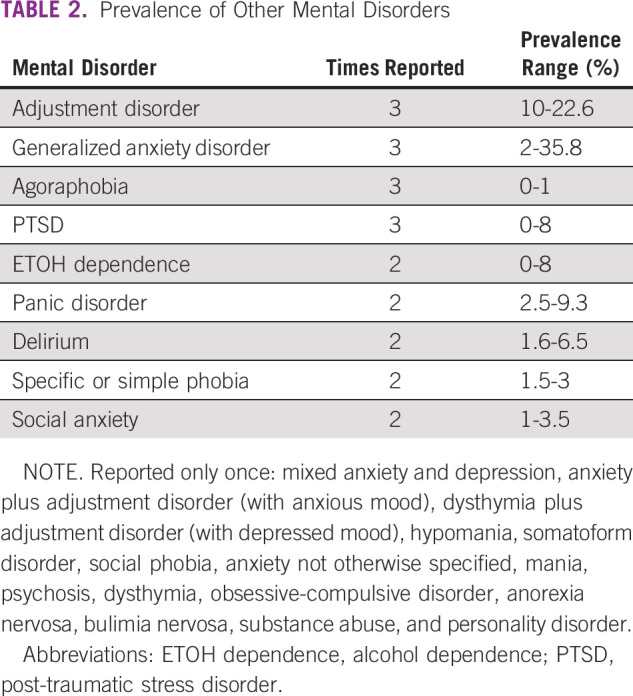
Prevalence of Other Mental Disorders

Associated factors of depression and/or anxiety symptoms were examined by 30 studies, with 27 studies identifying statistically significant associated factors. These factors are listed in Table 1. In terms of biologic factors, 12 studies found a significant association between advanced cancer stage and mental disorders^15,17,21,22,30,31,36,37,39,40,42,43,45,46,49^ with an odds ratio as high as 14.42.^40^ With regard to physical symptoms, cancer pain was found to increase the likelihood of depressive and anxiety symptoms.^40,45,46^ Higher rates of mental disorders or greater severity of symptoms were observed among a range of cancer sites and cancer types,^17,28^ with none consistently predominating. There were significant sex differences referenced in several studies. Being female was found to correlate with an increased likelihood of experiencing depression,^28,30^ as well as any form of mental illness,^20,31^ but findings were mixed with regard to anxiety.^28,30^ Similarly, mixed findings were reported in the inter-relationship between age and symptoms of mental illness in patients with cancer.^28,40^

There were several reports on the impact of marital relationships on the occurrence of emotional symptoms in patients with cancer. In both Nigeria and the Philippines, patients with cancer were more likely to report depression or depressive symptoms if they were unmarried or widowed.^15,49^ The inter-relationship between the home environment and overall emotional symptoms was also explored, with a tense home environment,^28^ absence of family support,^40^ and poor social support^49^ leading to a significant likelihood of patients with cancer developing emotional symptoms.

Several studies captured socioeconomic disadvantage as a risk factor for the development of emotional symptoms in cancer sufferers in LLMICs. Specifically, higher rates of depressive and anxiety symptoms were found among patients with cancer of a lower educational level.^17,21,24,28,42^ Higher levels of anxiety^43^ and depressive symptoms^17^ were found among patients with lower incomes or with low socioeconomic status.^22^ Contradicting this, a single study reported higher rates of depression in patients with cancer of middle or high socioeconomic status or a higher level of education.^32^ Interestingly, two studies found agricultural workers suffering from cancer to experience higher levels of depressive symptoms compared with office workers with cancer.^17,24^

Four studies examining the effectiveness of interventions addressing depression or anxiety among patients with cancer in LLMICs were identified. Two studies were based in India^50,51^ and two in Nigeria,^52^ with three of the four published since 2013.^50,52,53^ The study characteristics and findings are summarized in Table 3. Three randomized control trials^50,51,53^ and one open trial of interaction^52^ were conducted. The samples were highly heterogeneous with a range of cancer sites and stage of treatment. In one study, the presence of a current major mental disorder was an exclusion criterion.^51^

**TABLE 3:**
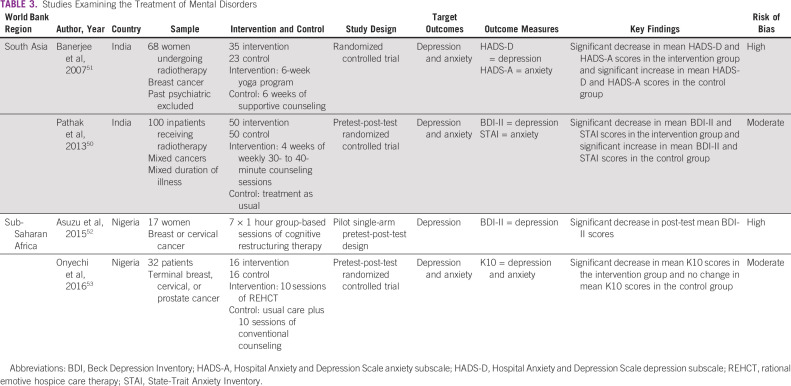
Studies Examining the Treatment of Mental Disorders

All four treatment studies were nonpharmacologic. Three studies measured the outcomes of psychotherapy treatments: four weeks of weekly counseling sessions compared with treatment as usual,^50^ 10 sessions of rational emotive hospice care therapy compared with conventional counseling,^53^ and seven sessions of cognitive restructuring therapy with no comparison group.^52^ A fourth study measured the outcome of a yoga program compared with supportive counseling.^51^

The four studies assessed the effect of the intervention on mean depression and/or anxiety symptoms as measured by the HADS, State-Trait Anxiety Inventory, Beck Depression Inventory II, or K10. No effect sizes were reported. Three studies used both depression and anxiety symptom rating scale scores,^50,51,53^ whereas one assessed the effect on depression symptom scale scores only.^52^ Following the psychotherapy intervention, three studies found a significant decrease in depression and anxiety symptom scale scores.^50,52,53^ The yoga program was found to be superior to the control group, which had received supportive counseling.^51^ The same study also found that the control group displayed an increase in depression and anxiety symptom scale scores.^51^

## DISCUSSION

This meta-analysis produced pooled prevalence estimates of depressive and anxiety disorders among patients with cancer living in LLMICs. When interview-based tools were used, the prevalence of DSM-IV–defined or ICD-10–defined major depression and anxiety disorders was 22% and 18%, respectively. In general, the estimates are higher than those previously reported in meta-analyses of interview-based studies examining the prevalence of depression or anxiety among patients with cancer. Mitchell et al^4^ found a pooled prevalence of 16.3% for major depression and 10.3% for anxiety, whereas Krebber et al^5^ arrived at a pooled prevalence of 14% for major depression. The discrepancy in the prevalence rates could be explained in part by the fact that the majority of studies included in the meta-analyses by Mitchell et al^4^ and Krebber et al^5^ were conducted in upper-income countries. The meta-analysis by Yang et al^54^ was restricted to studies based in China and spanned the period that China was classified as a lower-middle–income country and after its reclassification as an upper-middle–income country. Yang et al^54^ identified prevalence rates for depression and anxiety, as defined by clinical diagnosis, of 47.49% and 44.93%, respectively.

When caseness was defined by self-report tools, this review found a crude pooled prevalence for depression and anxiety ranging between 6.7%-93.3% and 5.6%-89.2%, respectively. For the acute phase of illness, Krebber et al^5^ found a pooled prevalence of 27% for depression diagnosed by self-report instruments and Yang et al^54^ found a pooled prevalence of 58.11% for depression and 51.74% for anxiety diagnosed by self-report instruments. However, both combined the results from disparate self-report instruments^5,54^ or identical self-report instruments but with disparate cutoffs, so comparisons are unable to be drawn.^54^

With regard to the general population in LLMICs, data from the 2017 Global Burden of Disease Study indicate a 12-month prevalence estimate for DSM-IV–defined or ICD-10–defined depressive disorders, including dysthymia, of 2.87% in lower-income countries and 3.24% in LLMICs.^55^ The 12-month prevalence of anxiety disorders was estimated to be 3.17% in lower-income countries and 3.36% in LLMICs.^55^ Both are substantially lower than the estimates identified in this meta-analysis.

This review examined the prevalence of other mental disorders in patients with cancer in LLMICs. In the broader global literature, mental disorders among patients with cancer, other than major depression and anxiety, generally appear to be less well-studied. However, meta-analysis of interview-based studies by Mitchell et al^4^ found a pooled prevalence for adjustment disorder of 19.4%. Meta-analysis by Abbey et al^56^ examined cancer-related PTSD and found that prevalence rates varied depending on assessment method, with prevalence rates ranging from 5.1% for interview-based PTSD to 11.2% for PTSD symptom clusters on the basis of self-report tools. A previous meta-analysis found that comorbid substance abuse rates in cancer ranged from 2% to 35%.^57^

This systematic review identified a broad range of reported associated factors contributing to an increased likelihood of experiencing emotional symptoms. Among them, the most consistently noted were advanced disease followed by a low level of education. Both factors were also reported in the systematic review by Niedzwiedz et al,^58^ which examined depression and anxiety among patients with cancer worldwide.

No published reports were found examining pharmacotherapy for the treatment of mental disorders among patients with cancer living in LLMICs. Multiple previous reviews have examined the evidence for this across the global literature. The findings are equivocal, with several meta-analyses finding some evidence of benefit,^59-63^ and others finding no clear evidence of benefit.^64,65^ Nonpharmacologic interventions evaluated in this review included psychologic therapies and yoga, a complementary therapy. In keeping with the current literature,^59,66^ this review found that psychologic interventions significantly improved depressive or anxiety symptoms. The superior outcomes of the group randomly assigned to yoga compared with counseling were consistent with the findings in the review by Cramer et al.^67^

To our knowledge, this review is the first to evaluate studies of mental disorders in patients with cancer in LLMICs and to establish a benchmark pooled prevalence of depressive and anxiety disorders. The current review was conducted in line with PRISMA guidelines, used a registered protocol, involved a broad search strategy, and performed screening in duplicate. The risk of bias was assessed using a validated tool, and sensitivity analyses were conducted. These steps confirmed that most studies included were of a low to moderate risk of bias.

This investigation has several shortcomings, and the results should be interpreted with caution. The limits imposed on the search had the potential to introduce bias, although the funnel plots indicate that relevant studies were not overlooked. The number of studies included was relatively small and used varying methodologies. The high heterogeneity of the pooled study populations further limits the generalizability of the results. The individual studies might have been prone to selection bias with most of the studies conducted in major cancer centers. Many LLMICs lack universal health coverage, and intracountry health inequalities can exist across socioeconomic, geographic, sex, racial, and ethnic lines. At least one study excluded participants with a prior mental disorder, and almost all the prevalence studies used a cross-sectional design. Finally, no causal relationship can be established between cancer morbidity and mental illnesses. Although previous literature has reported that rates of depression tend to peak in the acute period during treatment,^5^ it was noted that time since diagnosis of cancer and onset of emotional difficulties varied widely in the included studies. It is of note that the included treatment studies had relatively small numbers and the duration of follow-up was short with any sustained longer-term benefits not mentioned.

Several caveats should be noted around the process of diagnosis. Only a minority of studies used validated interview-based tools, which are considered the gold standard to identify depressive and anxiety disorders. Although measurement tools were previously validated in English, the translated versions often lacked local validation and cultural adaptation. It is possible that the frequent use of self-report tools to identify depressive and anxiety conditions might have led to an overestimation of prevalence rates. This flaw may explain the variation in rates when measured by observer-rated versus self-rated methods. We encourage future researchers to be selective in their choice and use of self-report tools, which are largely validated as screening, not diagnostic tools.

Of the 84 countries classified as LLMICs in 2018, only 15 were represented in our review. Many LLMICs, ethnic groups, and minority populations remain unrepresented. Future research could examine these populations or investigate broader psychologic distress in response to cancer among people living in LLMICs. Suicide was beyond the defined scope of this study and warrants exploration given the higher rate of suicide in patients with cancer.^68^ The dearth of treatment studies identified in this review highlights opportunities for future research, such as assessing antidepressants from the WHO essential medications list or exploring culturally appropriate psychosocial interventions. More broadly, there is a need for increased mental health research in LLMICs. Globally, mental health research is not funded proportional to the burden of disease^69^; furthermore, the distribution of this research is inequitable, with negligible funding for central Asia, the Middle East, and Africa.^69^

The International Psycho-Oncology Society International Standard on Quality Cancer Care, endorsed by International Psycho-Oncology Society and the Union for International Cancer Control, states that “*Psychosocial cancer care should be recognised as a universal human right; Quality cancer care must integrate the psychosocial domain into routine care; Distress should be measured as the 6th Vital Sign after temperature, blood pressure, pulse, respiratory rate and pain*.”^70^ However, psychosocial care is often not an established part of cancer care. National Cancer Control Plans (NCCP) are government plans that guide cancer prevention and management for each country. At present, NCCPs are being developed and updated in individual LLMICs to address the growing cancer burden. Not all NCCPs include psychosocial cancer care,^71^ and some LLMICs do not yet have an NCCP.^72^ This review reveals the existence of a substantial burden of disease and the importance of the greater recognition of psychosocial needs by clinicians in LLMICs. Furthermore, it adds weight to the importance of psychosocial care being consistently included in the NCCPs of LLMICs, with the aim of psychosocial care becoming an integral part of routine care. Future research could examine models of delivery, as well as effective and culturally acceptable treatment options, which could be integrated into comprehensive cancer care.

Once previously overlooked, mental disorders are now acknowledged as common and affect culturally and ethnically diverse people across the globe. This review suggests that considerable levels of mental disorder exist among patients with cancer in LLMICs. With the development of national cancer care plans in LLMICs, there is the opportunity to embed and integrate mental health care into cancer care services. Although interventions that target cancer-related mortality are essential in resource-limited settings, addressing the unmet mental health burden could improve survival rates and may be critical for improving quality of life.
